# Feasibility and safety of repeated 23% hypertonic saline boluses for electrical impedance tomography contrast enhancement in anaesthetised horses

**DOI:** 10.3389/fvets.2026.1789443

**Published:** 2026-06-15

**Authors:** Kieran Smith, Anthea Raisis, Fernando Moreno-Martinez, David Byrne, Martina Mosing

**Affiliations:** 1 Murdoch University School of Veterinary and Life Sciences, Perth, WA, Australia; 2 Anaesthesiology and Intensive Care, Clinical Centre for Small Animal Health and Research, Clinical Department for Small Animals and Horses, University of Veterinary Medicine Vienna, Vienna, Austria

**Keywords:** baseline impedance drift, contrast-enhanced EIT, haemodynamic effects, large-animal EIT, pulmonary perfusion, thoracic impedance

## Abstract

Hypertonic saline (HS) has been used to enhance electrical impedance tomography (EIT) cardiac-related signals associated with pulmonary perfusion in other species, but its suitability and physiological effects in horses are unknown. This exploratory study aimed to determine whether intravenous administration of 100 mL of 23% HS produces a detectable change in thoracic impedance in anaesthetised horses, and to evaluate the physiological effects of repeated bolus administration. Six adult horses were anaesthetised twice using total intravenous anaesthesia. Hypertonic saline 23% was administered via a jugular catheter every 15 min (T15-T60). End-expiratory lung impedance (EELI), tidal impedance variation (TIV), heart rate (HR), and mean arterial pressure (MAP) were recorded at pre-injection, post-injection and end-sample timepoints. Plasma sodium (Na^+^) and chloride (Cl^−^) concentrations were measured before each injection and 15 minutes after the final injection (T75), while urinary fractional excretion (FE) was calculated at T15 and T75. Changes over time were assessed using repeated-measures ANOVA, and FE was compared using paired t-tests. Following HS injection, there was a significant decrease in EELI (*p* < 0.01), and unanticipated haemodynamic effects were observed, including a significant decrease in MAP (*p* < 0.01) and increase in HR (*p* < 0.01), with hypotension (MAP below 70 mmHg) occurring in 62% of post-injection measurements. TIV decreased significantly (*p* = 0.02), and alterations in breathing pattern occurred in 24/47 post-injection sample times, including transient tachypnoea, expiratory pauses, and crown-like breaths. Plasma Na^+^ and Cl^−^ increased significantly over time (both *p* < 0.01), and FE of Na^+^ and Cl^−^ increased between T15 and T75 (*p* = 0.01 and *p* = 0.02, respectively). Injection of 100 ml of 23% HS reduced EELI, supporting potential use for enhancing perfusion-related EIT signals. Repeated boluses produced clinically relevant decreases in MAP. Concurrent changes in TIV and breathing pattern may complicate interpretation of EIT-derived ventilation-perfusion relationships. Accordingly, repeated 23% HS boluses cannot currently be recommended for EIT contrast enhancement in horses. Further work is required to determine whether single-bolus or lower-concentration protocols provide adequate signal enhancement with less physiological disturbance.

## 1 Introduction

Electrical impedance tomography (EIT) is a non-invasive, radiation-free imaging modality that has been used to provide real-time information on the global and regional distribution of ventilation in several species in both awake and anaesthetised states (1). In recent years, increasing evidence has supported the use of EIT to identify cardiac-related signals (CRS) in animals (2–9). In anaesthetised horses, EIT has been able to detect the heart rate with excellent agreement with invasive blood pressure trace (10). Hypertonic saline and EIT was utilised to capture first-pass impedance changes across the thorax of anaesthetised white rhinoceros, allowing construction of regional perfusion maps that demonstrated distribution of blood between dependent and non-dependent regions (2).

Blood has low electrical impedance (i.e., high electrical conductivity), thus the decrease in thoracic impedance during systole as pulmonary vessels filled with blood is small (11). These CRS are imperceptible when compared with those generated by tidal ventilation and are often obscured by motion artefact from the beating heart. Various methods - including apnoea-induced signal separation, ECG gating, and frequency filtering - have been used to isolate perfusion signals, but signal quality remains limited (4, 8, 9, 12, 13). To enhance detection, a bolus of hypertonic saline (HS) can be used as a conductive enhancing agent. When injected rapidly into a central vein, the bolus passes through the right heart and pulmonary circulation. Because hypertonic saline contains a high concentration of free ions (Na^+^ and Cl^−^), it transiently increases electrical conductivity of the blood within the pulmonary vessels. This produces a transient, localized decrease in thoracic impedance which can be quantified by assessing the decrease in end-expiratory lung impedance (EELI) as the HS passes through the pulmonary vessels. The impedance then returns to baseline as the HS exits the pulmonary circulation and becomes diluted by blood within the larger vessels. These rapid, region-specific impedance decreases with high temporal resolution can be recorded and evaluated using special software. The magnitude and timing of the decreased impedance in each pixel correspond to regional pulmonary blood flow, allowing reconstruction of an image related to pulmonary perfusion from the spatial distribution of this first-pass conductivity change (1, 14, 15).

A rapid injection of hypertonic saline (HS) is required to ensure adequate temporal enhancement. In the medical literature, injection durations of less than 2 s have been recommended to maximise contrast enhancement (14). However, there is currently no consensus regarding the optimal injection speed, volume, or saline concentration for this purpose in any species. Saline solutions of variable concentration (3%−23%), administered in volumes ranging from 10–130 ml over 4–20 s, have been reported across multiple species (2, 3, 6, 8, 14). In anaesthetised pigs, a 10 ml bolus of 5.85% HS was administered (rate not specified) and this concentration was identified as optimal for inducing conductivity changes, producing high signal strength and image quality (4). This dose corresponded to approximately 0.25 ml kg^−1^ of HS and a sodium load of approximately 1 mmol kg^−1^ in pigs with a mean body weight of 41 ± 5 kg (4). Based on this data and a preference to minimise injection volume, doses of 100 ml of 23% HS were selected for the present study. However, the thoracic anatomy and pulmonary circulatory volume of horses differ substantially from those of smaller species and thus it is important to determine if this dose is sufficient to produce an adequate impedance signal.

As repeated measurements are frequently required during research to assess responses to treatments, repeated administration of HS may be necessary when EIT is used to evaluate changes in pulmonary perfusion over time. Consequently, the physiological effects of repeated administration of 23% HS must be determined.

The reported effects of HS in horses are currently limited to its use for volume resuscitation. Hypertonic saline expands plasma volume by shifting fluid from the intracellular and interstitial spaces into the vascular space. In conscious horses, a single dose of 5 ml kg^−1^ of 7.5% HS (given over 30 min) increased plasma volume by 12.3%, and in anaesthetised horses 4 ml kg^−1^ of 7.2% HS (over 10 min) significantly elevated blood pressure and systemic vascular resistance (SVR) (16, 17). However, these studies administered HS as relatively slow infusions, whereas the rapid bolus injection required for EIT contrast enhancement is delivered over seconds. As the rapid injection rate may influence the haemodynamic response to hypertonic saline, the cardiovascular effects of rapid bolus administration on plasma volume, MAP, and HR in horses remain uncertain. Furthermore, it is not known what effect repeated injections of 23% HS will have on cardiovascular function and electrolyte balance.

The first aim of this study was to determine whether intravenous administration of 100 ml of 23% HS would produce a detectable change in thoracic impedance, as measured by EIT, in anaesthetised horses. The second aim was to evaluate the effects of repeated HS injections on plasma electrolyte concentrations, urinary fractional excretion of electrolytes, and cardiorespiratory variables. It was hypothesised that each injection of 23% HS would cause a visible and quantifiable decrease in end-expiratory lung impedance (EELI). Based on the known plasma volume-expanding effects of hypertonic saline described above, it was also hypothesised that repeated injections would increase MAP, plasma sodium and chloride concentrations and urinary fractional excretion.

## 2 Materials and methods

Data for this study were collected as part of a larger study evaluating the effects of airway management (endotracheal tube [ETT] vs. facemask [MASK]) on breathing pattern and distribution of ventilation in anaesthetised horses, assessed using electrical impedance tomography and complementary respiratory measurements (18). The sample size of six horses was determined *a priori* for the primary outcomes of the parent study; no separate sample size calculation was performed for the hypertonic saline analysis. All procedures were approved by the Murdoch University Animal Ethics Committee (protocol R3294/20).

Each horse was anesthetised twice for this experimental, randomised cross-over study. Animals were allowed to breathe room air spontaneously via ETT or MASK, considered the treatment, for 30 min, followed by the other treatment for an additional 30 min. A coin was used to assign the randomized treatment order for the first anaesthesia. During a second anaesthesia 1 month later, the treatment order was reversed.

### 2.1 Animals

Six male horses (Standardbred *n* = 4, Thoroughbred *n* = 2), were recruited from the Murdoch University teaching herd with a median age of 13 years (range 8–21 years), a median bodyweight of 553 kg (range 487–634 kg), and a median BCS of 5/9 (range 4–7/9). Horses were clinically healthy (ASA I) as per history and clinical exam. Horses were moved from the teaching herd into irrigated paddocks in pairs at least 48 h before the experiment. These paddocks offered unrestricted access to grass and water.

### 2.2 Anaesthesia

On the day of the experiment, the horse was moved into stocks and a 14-gauge catheter (BD Angiocath IV Catheter, BD ANZ, Melbourne, Australia) was placed aseptically in the right jugular vein after local infiltration block of lidocaine (Lignocaine Ilium, Troy Laboratories Pty Limited, Australia) superficial to the right jugular vein. Xylazine 0.5 mg kg^−1^ (Ilium Xylazil-100, Troy Animal Healthcare, Glendenning NSW, Australia) was administered intravenously (IV). Another 14-gauge catheter (BD Angiocath IV Catheter, BD ANZ, Melbourne, Australia) was placed aseptically into the left jugular vein.

The horse was moved into the induction box and additional xylazine 0.7 mg kg^−1^ IV was administered. 3 min later, anaesthesia was induced with ketamine (Ketamine Ceva, Ceva Animal Health Pty Ltd, Australia) 2 mg kg^−1^ IV and diazepam (Diazepam Ilium, Troy Laboratories Pty limited, Australia) 0.1 mg kg^−1^ IV. Once recumbent, the horse was hoisted onto a padded surgical table and positioned in right lateral recumbency. Anaesthesia was maintained with an IV infusion of ‘*triple drip'* comprising xylazine 1 mg kg^−1^ h^−1^, ketamine 2 mg kg^−1^ h^−1^, and guaiphenesin (Guaiphenesin Ilium, Troy Laboratories Pty limited, Australia) 75 mg kg^−1^ h^−1^. The drug mixture was administered at 1.56 mL kg^−1^ h^−1^ for 15 min and 0.78 ml kg^−1^ h^−1^ for the remainder of the anaesthesia. Anaesthetic depth was monitored constantly by an experienced anaesthetist (AR) and additional xylazine 0.25 mg kg^−1^ IV and ketamine 0.5 mg kg^−1^ IV were administered to increase the depth of anaesthesia if required to prevent movement. Horses breathed room air spontaneously for the duration of anaesthesia. Oxygen supplementation was provided when two consecutive PaO_2_ measurements taken 15 min apart were below 60 mmHg. Oxygen supplementation was delivered at 10–15 L min^−1^ through the ETT or MASK via a customized large animal non-rebreathing system (19). Hartmann's solution 3 ml kg^−1^ h^−1^ IV (Hartmann's solution, Baxter Healthcare Pty Ltd, Australia) was administered through the right jugular catheter. Monitoring, beside that specified below, consisted of a lead II electrocardiogram (SurgiVet^®^ Smith Medicals, Minnesota USA) and pulse oximetry (Radical-7^®^ Masimo Corporation^®^, Irvine CA). Heart rate, respiratory rate, partial pressure of end-tidal CO_2_, systolic, mean, and diastolic arterial pressures, oxygen saturation of haemoglobin, and clinical signs of the depth of anaesthesia were manually recorded every 5 min for the duration of anaesthesia. After 60 min of anaesthesia, triple drip infusion was discontinued, the EIT belt and all catheters removed bar one jugular catheter for ongoing venous access, and the horse hoisted to a padded recovery box for unassisted recovery. 15 min after standing, the horses were walked back to the paddock and monitored for another 24 h before returning to the herd.

To investigate the effects of hypertonic saline, instrumentation included the placement of an EIT belt, an arterial catheter for measurement of blood pressure and electrolytes and insertion of a urinary catheter. The EIT belt was placed before anaesthesia was induced. At the level of the 6th intercostal space, the horse's hair coat was wetted with tap water and a low-conducting gel applied. A customized EIT belt (32 gold-plated washer electrodes, one plane) was placed and held in contact with the skin by application of a surrounding cohesive bandage (1). The position of the EIT belt was checked after being positioned on the table. The EIT electrode belt was connected to the EIT device (BBVet, SenTec AG, EIT branch, Switzerland), linked to a laptop with dedicated software to record EIT data applying an equine species-specific finite element model (BBVet SW, SenTec, Switzerland).

An arterial catheter was placed in the lateral metatarsal artery of the left (upper) hindlimb following positioning of the horse on the table. For measurement of blood pressure, the arterial catheter was connected to a transducer (Meritrans DTX Plus, MeritMedical, UT, US), which was positioned at the level of sternum and zeroed to atmospheric pressure. The transducer was then connected to the multiparameter monitor for recording of pressure waveform (Surgivet V9203; Smiths Medical, MA, USA). Blood was also sampled from the arterial catheter for measurement of serum creatinine, blood gases and electrolytes. A urinary catheter (Foley 24Fr 30cc, MILA International Inc, KY, US) was placed after positioning the horse for emptying the bladder during the anaesthetic period and collection of urine for calculation of fractional excretion of electrolytes.

### 2.3 Study timeline and data collection

**Figure 1:**
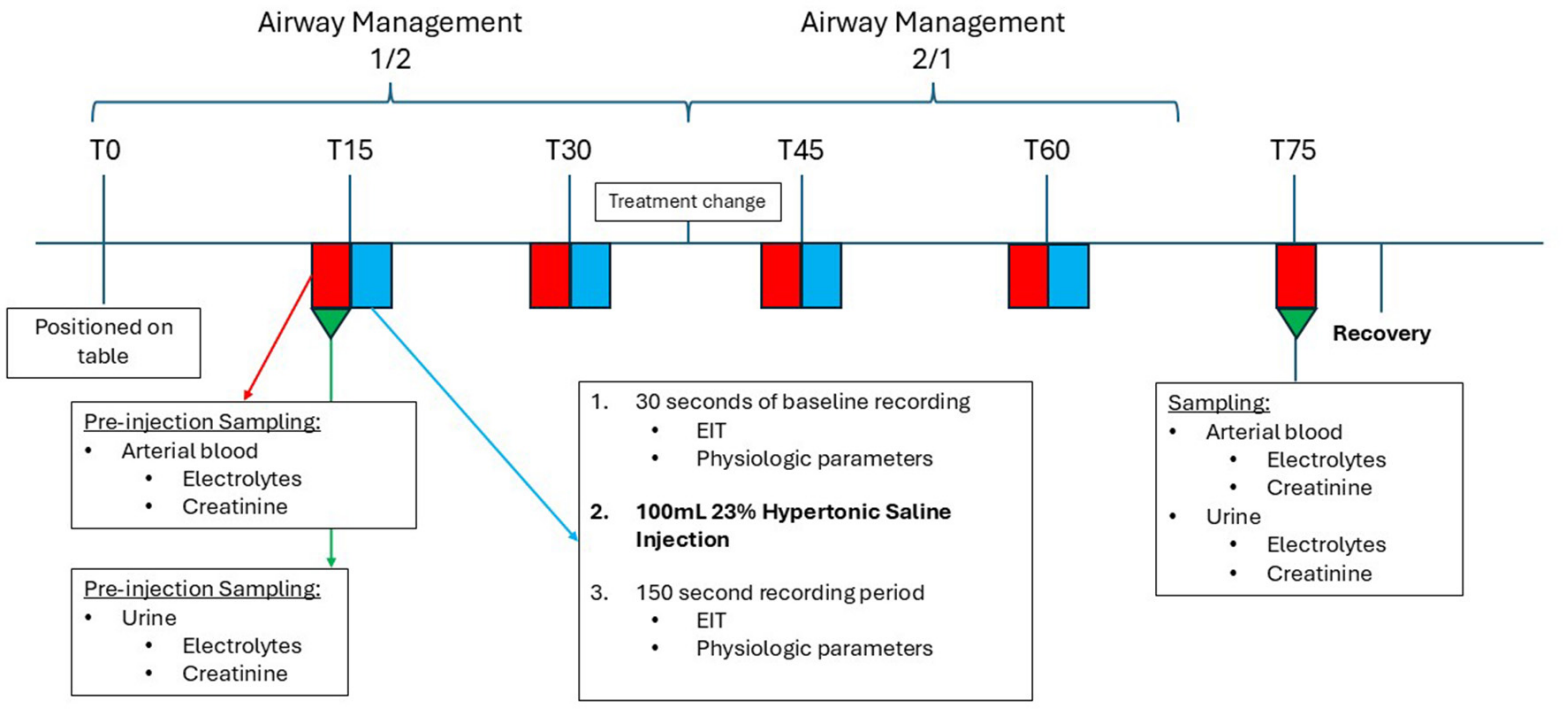
Study timeline. T0, 15, 30, 45, 60, 75 depict minutes elapsed since positioning the anaesthetised horse on the table. Airway management 1/2 and 2/1 refer to the order of airway techniques used during anaesthesia: 1/2 indicates endotracheal intubation (ETT) followed by facemask ventilation (MASK), whereas 2/1 indicates facemask ventilation followed by endotracheal intubation. Swapping of the management occurred between T30 and T45. The airway management order was randomly allocated. EIT, electrical impedance tomography.

Data collection for each study occurred at 15 (T15), 30 (T30), 45 (T45), 60 (T60), and 75 (T75) minutes after positioning the horse on the table (T0). For the purposes of the airway study, either orotracheal intubation was performed using a 26 mm ID ETT (ETT) or a tight-fitting facemask was placed over their muzzle and nostrils (MASK) at T0. After data collection at T30, horses assigned ETT were extubated and the face mask positioned over the muzzle, while horses assigned MASK had the facemask removed and were orotracheally intubated (Figure 1).

Each horse received 100 ml intravenous boluses of 23% HS administered into the left jugular vein at 15 (T15), 30 (T30), 45 (T45), and 60 (T60) minutes. During the first anaesthetic 100 ml of 23% HS was administered over 10 s. Due to the unexpected and marked cardiorespiratory effects observed (see results), the same dose was administered over 20 s during the second anaesthetic. Injections were performed manually using syringes, with the operator timing the injection using a stopwatch to ensure the target injection duration was maintained as consistently as possible.

EIT data was recorded continuously from 30 s prior to each injection until 150 s after the start of injection. Representative screenshots from BBVet showing temporal changes in EELI were also taken.

Arterial blood was collected immediately prior to each 23% HS injection (T15, T30, T45, T60) and again 15 min after the final HS injection (T75). 1 ml of blood was collected directly into heparinised syringes (Radiometer Medical ApS, Brønshøj, Denmark) for measurement of blood gases and electrolyte concentrations (Na^+^, K^+^, Cl^−^, Ca^2+^). 5 ml of blood was also collected and placed in a plain blood tube for measurement of serum creatinine and serum electrolytes. Urine samples (20 ml) were collected at T15 and T75 for measurement of electrolyte concentrations (Na^+^, K^+^, Cl^−^) and urine creatinine concentration. The urine sample collected at T15 was performed prior to the first injection of 23% HS and included urine present prior to induction and during the first 15 min of anaesthesia. Between T15 and T60 the bladder was allowed to drain continuously, and urine was discarded. At T60 the catheter was occluded to allow accumulation of urine over the 15 min following the last injection of HS (Figure 1).

Two video cameras (GoPro Inc., San Mateo, California, USA) continuously recorded all monitoring equipment including the BBVET data and the multiparameter anaesthetic monitor (Surgivet V9203, Smiths Medical, MA, USA). A digital clock displaying hours, minutes and seconds was positioned within the field of view of both cameras and served as a reference timestamp. These recordings were used to retrospectively synchronise data collection with each hypertonic saline injection.

### 2.4 Data analysis

#### 2.4.1 EIT data processing

Electrical impedance tomography recordings were acquired in real time using BBVet software (BBVet SW, SenTec, Switzerland), and raw impedance data were saved as .ZRI files. Data were analysed in IBEX (Swisstom AG) using a species-specific finite element mesh incorporating the anatomic boundaries of the equine thorax and lungs. Functional EIT images were reconstructed, and global impedance signals extracted. For each timepoint, a 150-s recording was obtained, comprising 30 s prior to and 120 s following the start of hypertonic saline injection. Impedance data were exported from IBEX into Microsoft Excel (Microsoft Corp., USA) for quantitative analysis.

**Figure 2:**
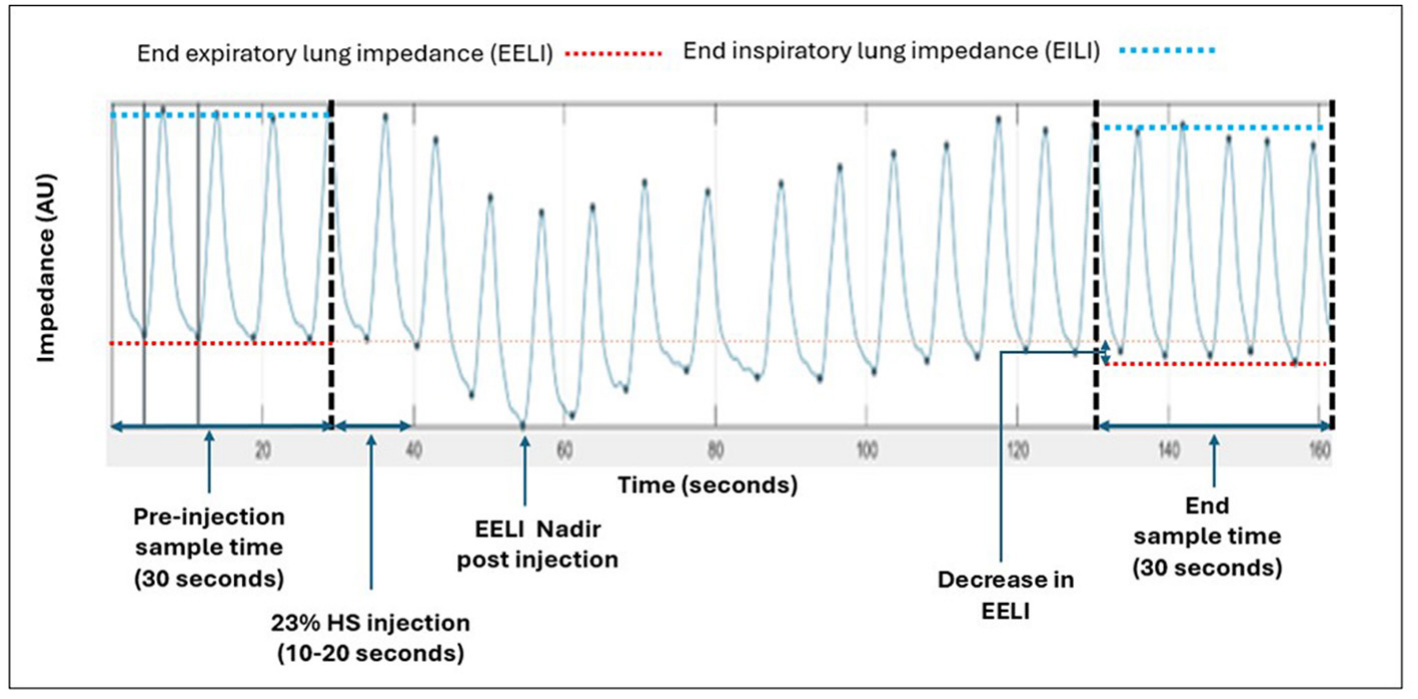
An example of the time-impedance curve and associated timepoints for data collection. The pre-injection EELI is immediately prior to injection with 23% HS. The nadir is the lowest value present following HS injection (typically within 20 s for EELI). The faint red dashed line is present to show the deviation from pre-injection ‘baseline' for EELI across timepoints. Blue dashed line indicates the end inspiratory lung impedance, with TIV being derived as the difference between this value and the EELI. EELI, end expiratory lung impedance; HS, hypertonic saline; TIV, tidal impedance variation.

End-expiratory lung impedance (EELI) was determined from the end-expiratory level of the global impedance curve, with the nadir defined as the lowest post-injection value (Figure 2). Tidal impedance variation (TIV) was calculated as difference between the mean end-inspiratory impedance and the start-inspiratory impedance (1). For both EELI and TIV, the pre-injection value was calculated as the average of 30 s of data immediately preceding injection, and the end-sample value was calculated from the final 30 s of each recording (Figure 2). As the horses were breathing spontaneously, the number of breaths per sampling window ranged from three to six.

The breath-by-breath changes were visually inspected using the BBVet software (BBVet SW, SenTec, Switzerland). Changes in pattern of breathing following 23% HS injection were also assessed from the EIT-derived impedance traces. Alterations in breathing pattern were predefined by the authors, relative to the pre-injection baseline for each individual horse. Events were predefined as tachypnoea (series of breaths at rate more than twice the pre-injection rate), expiratory pause (time between breaths >10 s), stacked (two superimposed inspiratory peaks of descending amplitude) or crown-like (two or more superimposed inspiratory peaks of similar amplitude) breaths. The number of altered breathing events within the 2-minute window following each HS bolus was noted. Patterns that did not differ from the pre-injection baseline were classified as normal and not recorded as altered events.

#### 2.4.2 Cardiovascular function

Cardiovascular data (heart rate, systolic, mean, and diastolic arterial pressure) were extracted from the video recordings of the multiparameter monitoring screen. For each sample time, the measurement of each variable obtained pre-injection, post-injection (peak or nadir), and at the end of the sample period were extracted. Each measurement represented an average of 10 s. Nadir values were the lowest 10 s average in the recording period following HS injection, and peak values represented the highest 10 s average following HS injection. The timing of the peak or nadir following end of injection was also noted.

#### 2.4.3 Electrolytes

Plasma electrolyte concentrations ([Na^+^], [K^+^], [Cl^−^]) were measured with an ABL analyser (ABL90, Radiometer, Denmark). Serum blood samples were allowed to clot and then centrifuged at 1,358g for 10 min. Serum was then decanted and used for measurement of serum creatinine and electrolytes. Urine from each horse was centrifuged at 339g for 5 min to remove the sediment. The serum and urinary creatinine concentration and serum electrolytes were measured on the same day of collection using a Cobas Integra 400plus biochemistry analyser (Roche Diagnostics, Switzerland). Creatinine was measured using the Compensated Jaffe method (CREJ2, Roche Diagnostics, Switzerland). Fractional excretion (FE) of sodium, potassium, and chloride was calculated using the standard formula:


$$\begin{array}{l} FEx={\left(\frac{Ux^{*}Scr}{Sx^{*}Ucr}\right)}^{*}100 \end{array}$$

where Ux and Sx are the urine and serum concentrations of the electrolyte of interest, and Ucr and Scr are urine and serum creatinine concentrations, respectively.

#### 2.4.4 Statistics

Continuous variables were assessed for normality using the Shapiro–Wilk test. As the data met the assumption of normality, results are presented as mean with 95% confidence intervals (CI). Differences in response to 23% HS injection between sample times during anaesthesia 1 and anaesthesia 2 were assessed using a mixed-effects model with sample time and anaesthesia episode included as fixed effects and horse included as a random effect. Response was defined as percent change in EELI, TIV, MAP, and HR from pre-injection to nadir/peak post-injection. When a significant main effect or interaction was detected (*p* < 0.05), *post hoc* pairwise comparisons were performed using Tukey's multiple comparison test.

A repeated-measures (RM) ANOVA was used to determine whether pre-injection values changed over time by comparing measurements at T30, T45, and T60 with those at T15. For plasma electrolytes, a further comparison of T75 against T15 was made. Where significant differences were identified, Dunnett's *post hoc* test was applied. To assess the effect of each 23% HS injection on each outcome variable, a separate RM ANOVA compared the peak/nadir and end-sample values to the pre-injection with significant findings likewise explored using Dunnett's *post hoc*. Fractional excretion values, calculated only at T15 and T75, were compared using paired *t*-tests.

The frequency of altered breathing patterns during first and second anaesthesia were calculated as the number of sample times with altered patterns divided by total number of sample times in each anaesthetic period expressed as a percentage. For each frequency the Wilson-Brown intervals were calculated. Fishers exact test was performed to determine the effects of anaesthetic episode (and thus speed of injection) on frequency of altered breathing patterns.

All analyses were two-tailed, significance was set at α = 0.05, and statistical procedures were performed using GraphPad Prism v10 (GraphPad Software, USA).

## 3 Results

All six horses completed this exploratory study. For the parent study, each horse underwent two anaesthetic episodes separated by 4 weeks. Supplemental oxygen was required in two horses after T30 in both anaesthetic sessions, and two horses during one of their anaesthetics (one during the first and one during the second). The remaining two horses did not require oxygen supplementation. All horses recovered uneventfully.

Each injection of 23% HS corresponded to a mean (SD) dose of 0.18 (0.02) ml kg^−1^ body weight, equivalent to 0.72 (0.06) mmol Na^+^ kg^−1^ injected over 10 s during the first anaesthesia and 20 s during the second. Across the four boluses given during each anaesthetic episode, the cumulative load corresponded to a mean (SD) total of 2.9 (0.2) mmol Na^+^ kg^−1^ and 2.9 (0.2) mmol Cl^−^ kg^−1^.

Data was obtained from 48 data collection points across the two anaesthetic episodes. Electrical impedance tomography (EIT) variables were successfully recorded for 47 of these (23 during the first anaesthesia [10 s HS injection] and 24 during the second anaesthesia [20 sHS injection]). Data from one sample (T15 in Horse 1) was excluded due to failing electrodes during the measurement window.

The effect of 23% HS on percentage change did not significantly differ between any sample times during the first (10 s injection) and second (20 s injection) anaesthesia for EELI (*p* = 0.17), TIV (*p* = 0.13), MAP (*p* = 0.93) or HR (*p* = 0.29). The effect of 23% HS on the frequency of altered breathing patterns did not differ significantly (*p* = 0.77) between anaesthesia 1 (injection speed 10 s) and 2 (injection speed 20 s). For comparison of pre-injection values over time data from both anaesthetics was pooled resulting in four sample times: T15, T30, T45 and T60. To assess the effects of 23% HS, the pre-injection, nadir/peak and end-sample values from every sample time were pooled.

### 3.1 Impedance variables

**Figure 3:**
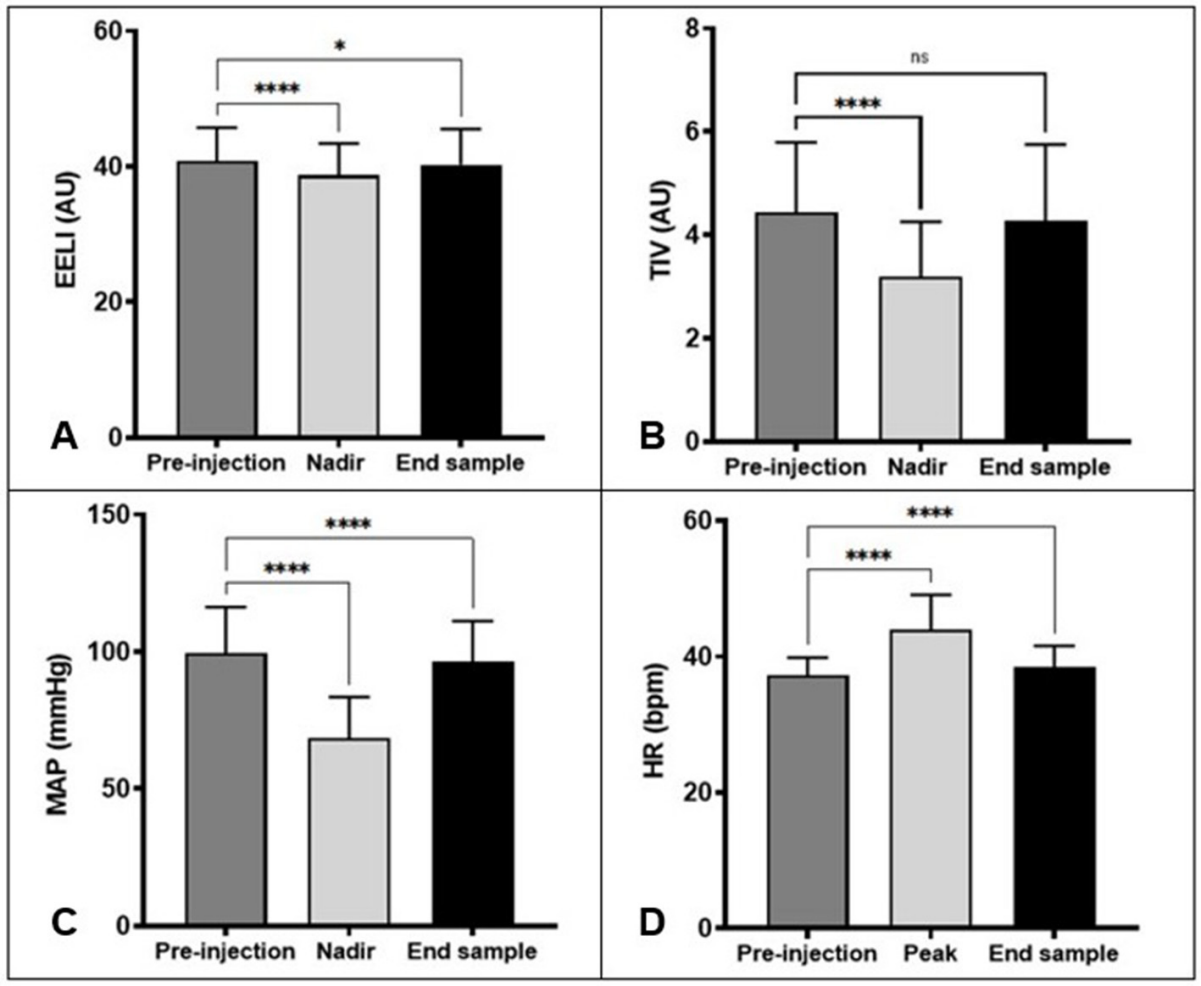
**(A)** Changes in end-expiratory lung impedance (EELI), **(B)** Tidal impedance variation (TIV), **(C)** Mean arterial pressure (MAP), and **(D)** Heart rate (HR) before and following injection of 23% hypertonic saline. The change in TIV presented is the change calculated for 23 sample times where no change in breathing pattern was observed. ‘Pre-injection' was measured immediately prior to injection, ‘end sample' was measured at the end of the sample time, and ‘nadir/peak' as the lowest or highest value following HS injection. Results are presented as the mean (95% confidence interval) of pooled data from T15, T30, T45, and T60 sample points in 6 horses anaesthetised twice each. Significant difference is indicated by *, with more asterisks indicating a stronger significance value [than 0.05].

**Figure 4:**
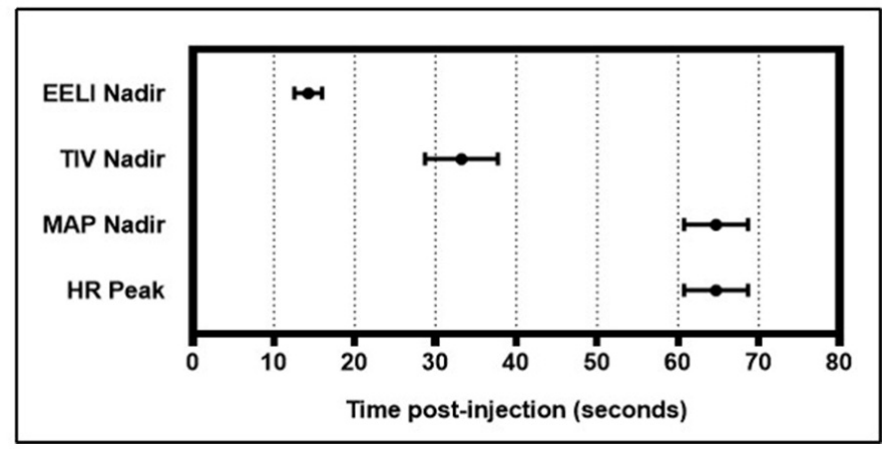
Mean (95% CI) time (seconds) from end of injection to the nadir/peak change of each variable. EELI, end expiratory lung impedance; TIV, tidal impedance variation; MAP, mean arterial pressure; HR, heart rate.

**Figure 5:**
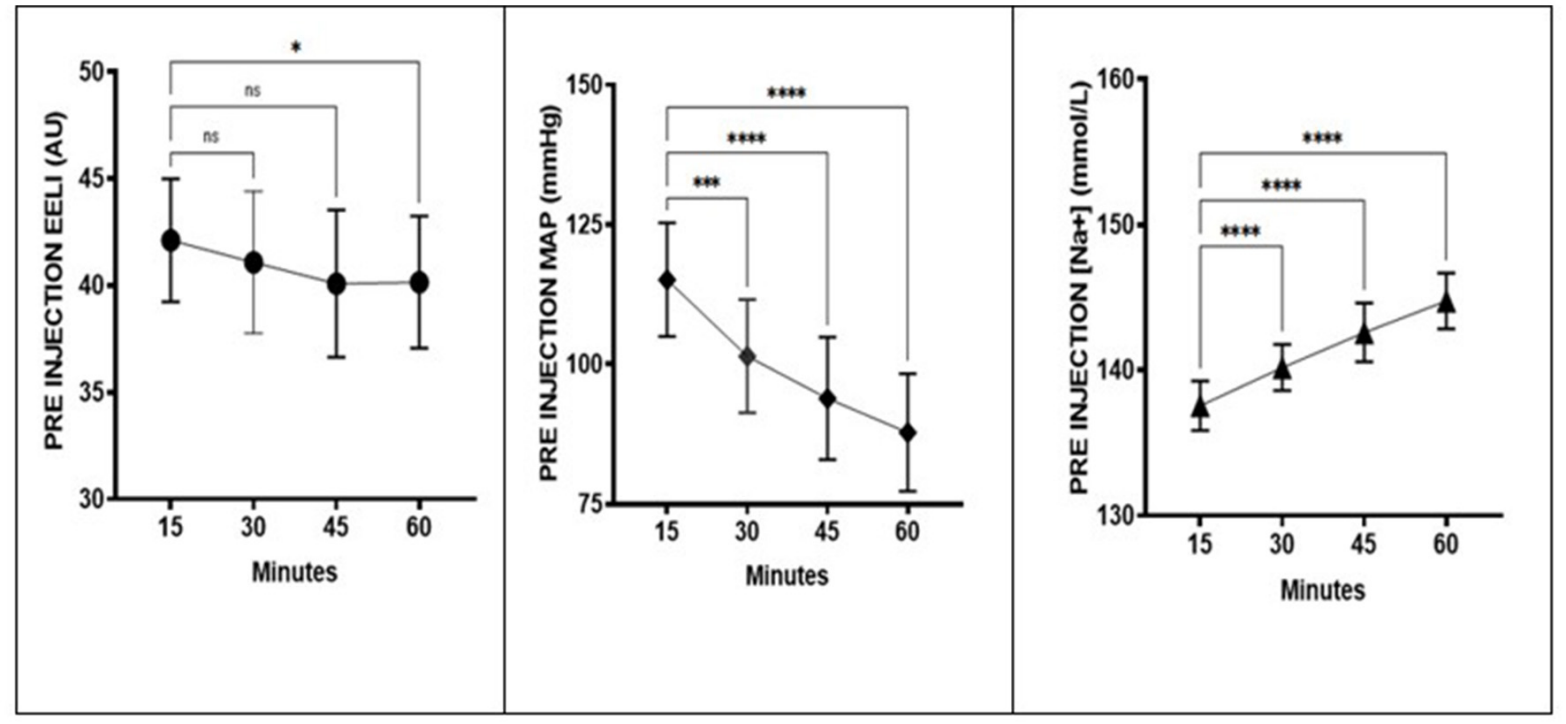
Mean (95% CI) for End Expiratory Lung Impedance (EELI), Mean Arterial Pressure (MAP), and arterial sodium concentration ([Na^+^]) at 15, 30, 45, and 60 min from 6 anaesthetised horses (47 sample times). The measurements at each sample time were recorded prior to injection of hypertonic saline. Significant difference from the first sample time of 15 min and subsequent sample times is indicated by *. More asterisks indicate a stronger significance value [than 0.05].

Following 23% HS injection, there was a significant (*p* < 0.01) decrease in mean [95% CI] EELI from pre-injection (40.8 [39.4–42.3] AU) to post-injection (38.7 [37.3–40.1] AU) (Figure 3A). The nadir of EELI occurred 14 (13–16) seconds after the injection (Figure 4). At the end of sample period, EELI (40.3 [38.7–41.8] AU) remained significantly lower than pre-injection (*p* = 0.04). Over time there was a significant decrease in pre-injection EELI between T45 (*p* = 0.03) and T60 (*p* = 0.01) and T15 (Figure 5, Table 1) but there was no difference between T15 and T30 (*p* = 0.41) (Table 1).

**Table 1:** Summary of mean (95% confidence interval) of variables recorded in anaesthetised spontaneously breathing horses.

Variable	T15	T30	T45	T60	T75
EELI (AU)	42.1 [39.2-45.0]	41.1 (*p* = 0.04) [37.8–44.4]	40.1 (*p* = 0.08) [36.6–43.5]	40.2 (*p* = 0.02) [37.1–43.2]	–
TIV (AU)	4.56 [3.68–5.44]	4.02 (*p* = 0.34) [3.30–4.75]	4.64 (*p* = 0.34) [3.69–5.60]	4.53 (*p* = 0.34) [3.58–5.49]	–
HR (bpm)	38 [36–40]	38 (*p* = 0.30) [36–40]	37 (*p* = 0.17) [35–39]	37 (*p* = 0.23) [35–39]	–
MAP (mmHg)	115 [105–125]	101 (*p* < 0.01) [91–112]	94 (*p* < 0.01) [82–105]	88 (*p* < 0.01) [77–98]	–
Plasma sodium (mmol/L)	137 [136–139]	140 (*p* < 0.01) [139–142]	143 (*p* < 0.01) [140–145]	145 (*p* < 0.01) [143–147]	147 (*p* < 0.01) [146–149]
Plasma potassium (mmol/L)	3.66 [3.55–3.78]	3.48 (*p* < 0.01) [3.24–3.61]	3.33 (*p* < 0.01) [3.19–3.46]	3.19 (*p* < 0.01) [2.95–3.43]	3.27 (*p* < 0.01) [3.13–3.40]
Plasma chloride (mmol/L)	102.2 [99.6–104]	105 (*p* < 0.01) [103–108]	106 (*p* < 0.01) [103–109]	109 (*p* < 0.01) [106–112]	112 (*p* < 0.01) [109–114]
FE sodium	0.89 [0.66–1.12]	–	–	–	9.83 (*p* = 0.01) [8.32–11.3]
FE potassium	54.3 [48.4–60.1]	–	–	–	54.6 (*p* = 0.48) [46.4–62.8]
FE chloride	2.78 [2.54–3.02]	–	–	–	12.4 (*p* = 0.02) [10.6–14.1]

The values at each time point represent the pooled values from two anaesthetic episodes performed 1 month apart. EIT data presented was recorded over 30 s prior to injection of 23% hypertonic saline at T15, T30, T45 and T60. Plasma electrolytes were measured immediately prior to each injection of 23% hypertonic saline at T15, T30, T45, T60 and 15 min after last injection (T75). Fractional excretion (FE) of electrolytes was calculated from serum and urine samples collected at baseline (T15) and 15 min after last injection (T75). Values are compared to baseline (T15) to determine significance (adjusted *p* value < 0.05).

**Figure 6:**
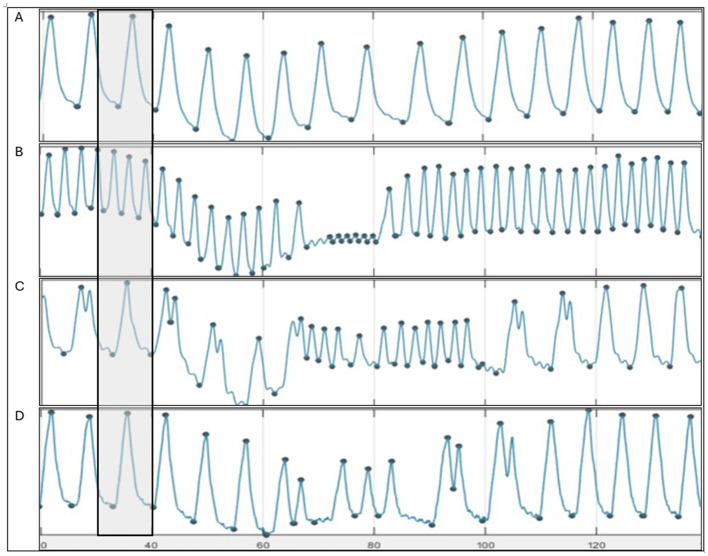
Examples of the pattern of breathing observed from visual assessment of breath-by-breath changes in impedance recorded in 6 anaesthetised horses. Each sample demonstrates the pattern before, during and following administration of 100 ml of 23% hypertonic saline. The shaded regions represent the time of 23% HS injection. **(A)** Normal pattern, **(B)** Tachypnoea, **(C)** Prolonged expiratory pause, **(D)** Stacked breaths.

Following 23% HS injection, there was a significant (*p* < 0.01) decrease in mean [95% CI] tidal impedance variation (TIV) across all 47 samples from pre-injection (4.44 [4.04–4.84] AU) to post-injection (3.19 [2.88–3.50] AU). In 24 of 47 samples, a change in pattern of breathing was observed (Figure 6). As altered breathing patterns could impact TIV, the remaining 23 samples without breathing pattern changes were analysed separately to determine whether the observed decrease in TIV was independent of breathing pattern. In the absence of altered breathing pattern mean [95% CI] TIV significantly decreased (*p* < 0.01) between pre-injection (4.78 [4.25–5.31] AU) and post-injection (3.65 [3.28–4.01] AU). The nadir of TIV decrease occurred at a mean [95% CI] of 33 (29-38) seconds following HS injection (Figure 4). At the end of sample time, TIV (4.69 [4.13–5.26] AU) was not significantly different from pre-injection values (*p* = 0.41) (Figure 3B). Pre-injection values of TIV did not significantly differ between T15 and other sample times (*p* = 0.24) (Figure 5).

### 3.2 Breathing pattern

Visual inspection of the EIT-derived impedance traces revealed three distinct alterations in breathing pattern following HS injections. A representative example of the normal and altered breathing patterns is presented in Figures 6A–D. Altered breathing patterns were observed in 24 of the 47 sample periods, comprising 11/23 (46 [28–68] %) during the first anaesthesia (10 s injection) and 13/24 (54 [34–74] %) for the second anaesthesia (20 s injection). During anaesthesia 1 and 2 respectively, 8/11 (73 [42–92] %) and 8/13 (62 [33–84] %) patterns were tachypnoea +/− expiratory pauses. The remaining altered patterns were crown-like breaths occurring in 3/11 (27%) samples during anaesthesia 1 (10 s injection) and 5/13 (38%) samples during anaesthesia 2 (20 s injection).

### 3.3 Changes in cardiovascular function

Following 23% HS injection, there was a significant (*p* < 0.01) decrease in mean [95% CI] MAP from pre-injection (100 [94–105] mmHg) to post-injection (69 [64–74] mmHg) (Figure 3C). The absolute decrease in MAP between pre-injection and post-injection was 31 (28-34) mmHg. The frequency of hypotension post-injection, defined as MAP < 70 mmHg, was 62% (20). In addition, MAP was < 60 mmHg post-injection in 21% of the sample times. Following 23% HS injection there was a concurrent and significant increase in mean [95% CI] HR (*p* < 0.01) from pre-injection (37 [36–38] bpm) to post-injection (44 [42–46] bpm) (Figure 3D). The mean [95% CI] nadir/peak of MAP/HR occurred simultaneously 65 [61–69] s after the end of the HS injection (Figure 4). At the end of each sample window, MAP (96 [92–101] mmHg and HR (38 [37–40] bpm) remained significantly different from pre-injection values (*p* < 0.01) (Figures 3C, D).

Over time the pre-injection MAP was significantly lower at T30 (*p* = 0.01), T45 (*p* < 0.01), and T60 (*p* < 0.01) when compared to T15 (Figure 5, Table 1). In contrast, pre-injection HR did not differ significantly between timepoints (*p* = 0.09) (Table 1).

### 3.4 Electrolytes and acid base variables

Plasma concentrations and urinary fractional excretion of electrolytes recorded prior to each 23% HS injection are summarised in Table 1. Both [Na^+^] and [Cl^−^] were significantly higher at T30, T45, T60, and T75 compared with T15 (all *p* < 0.01) (Table 1). Plasma [K^+^] was significantly lower at T30 (*p* = 0.01), T45 (*p* < 0.01), T60 (*p* = 0.01), and T75 (*p* < 0.01) than T15. Urinary fractional excretion of sodium and chloride increased significantly between T15 and T75 (*p* = 0.01; *p* = 0.02), whereas fractional excretion of potassium did not differ significantly between timepoints (*p* = 0.48).

## 4 Discussion

This study showed that 23% hypertonic saline consistently reduced end-expiratory lung impedance in anaesthetised horses, supporting the primary hypothesis. However, repeated bolus administration was associated with clinically relevant haemodynamic changes. Contrary to expectations, MAP decreased and HR increased after HS injection. Additional changes in tidal impedance variation and breathing pattern were also observed. Repeated boluses were associated with progressive increases in plasma sodium and chloride concentrations and their fractional excretion.

### 4.1 End expiratory lung impedance

The present study showed that in horses, 100 ml of 23% HS - equivalent to ~0.18 ml kg^−1^ - was sufficient to produce a rapid and reproducible decrease in end-expiratory lung impedance (EELI), indicating that 23% HS can act as an effective enhancing agent for detecting perfusion-related EIT signals in anaesthetised horses. This finding supports the primary hypothesis of the study and is consistent with reports in humans and other species where hypertonic saline has been used to enhance perfusion-related EIT signals (2–4, 6, 8, 14). The dose chosen for use in this equine study was extrapolated from information available in other species and the considerations for ease of manual injection. In our study 100ml of 23% HS was able to be administered over 10 (anaesthesia 1) and 20 (anaesthesia 2) seconds, both of which produced significant decreases in EELI.

### 4.2 Tidal impedance variation

A significant and unexpected decrease in tidal impedance variation (TIV) was evident following each injection of 23% HS. Changes in breathing pattern observed in 24 out of 47 samples plausibly explain, at least partly, this drop in TIV. However, TIV also decreased in samples where breathing pattern did not change. Another possible explanation for the decrease in TIV is a decrease in tidal volume, as these two variables have been previously shown to correlate (21). Another plausible reason for the decrease in TIV would be altered lung mechanics due to increased pulmonary blood volume secondary to hypertonicity-induced vasodilation (see below). This is supported by studies that demonstrate that increases in pulmonary blood volume or pulmonary arterial pressure reduce pulmonary compliance (22–24). Lastly, changes in impedance are measured in arbitrary units (15). It is possible that a transient but significant decrease in baseline impedance, due to HS-induced increased conductivity, may have altered the relative impedance change measure during breathing. Because spirometry was not performed and airway management interventions occurred concurrently, the relative contribution of altered respiratory mechanics and impedance-related measurement effects could not be determined. The return of TIV to pre-injection values by the end of the sample period suggests this was a transient effect; however, its physiological significance remains unclear.

### 4.3 Qualitative changes in breathing pattern

Alterations in breathing pattern occurred after HS injection in 24 of the 47 sample periods, characterised by tachypnoea, expiratory pauses, and, in some horses, crown-like breaths (Figure 6). These changes occurred after the transient decrease in EELI and were therefore temporally associated with systemic distribution of the HS bolus. Similar tachypnoea and expiratory time prolongation have been reported in rats receiving intra-arterial HS once osmolality exceeded ~340 mOsm kg^−1^, mediated by peripheral and central chemoreceptors (25). Although the sodium load in the present study was lower (~0.8 mmol; 1.6 mOsm kg^−1^), arterial osmolality was not measured immediately after injection, and a sodium-driven mechanism cannot be excluded.

The 23% HS dose used in the present study was selected to provide sufficient ionic load for contrast enhancement while limiting injection volume. This was extrapolated from anaesthetised pigs, in which 5.85% HS delivering approximately 1 mmol kg^−1^ sodium produced optimal conductivity changes (4). In horses, achieving a comparable sodium load with a lower-concentration solution would require a substantially larger volume, which could not practically be delivered within the short injection window required for temporal contrast enhancement. Accordingly, 23% HS was selected to allow rapid administration of a 100 ml bolus with a sodium load considered likely to generate a detectable impedance signal. After unexpected cardiorespiratory responses were observed with the initial 10-s injection, the injection duration was extended to 20 s. Within this limited comparison, slowing the injection did not appear to mitigate the observed responses. However, this exploratory study cannot separate the relative contributions of injection rate, concentration, sodium load, bolus volume, or the injection procedure itself. Further studies are required to determine whether lower-concentration HS or smaller sodium loads can preserve EIT enhancement, and to clarify the mechanism, repeatability and clinical relevance of the observed cardiorespiratory changes.

Because the respiratory changes were unexpected and occurred outside the predefined sampling schedule approved for this study, arterial blood gas sampling was not performed during these events, limiting the interpretation of the physiological and clinical impact of HS on gas exchange. However, mean PaO_2_ and PaCO_2_ at each timepoint prior to HS injection did not differ, suggesting a transient effect (18). These breathing pattern alterations are also likely to interfere with interpretation of ventilation-perfusion relationships when using EIT, supporting investigation of lower concentrations or alternative approaches for EIT perfusion assessment.

### 4.4 Quantitative changes in cardiovascular function

Contrary to our hypothesis, MAP transiently decreased while HR increased following each HS injection. The changes in MAP and HR occurred concurrently and consistently followed the reduction in EELI (Figure 4), suggesting a temporally linked physiological response. This response is contrary to the increased MAP and decreased HR reported when HS is administered for volume resuscitation in horses with a slower injection rate (26, 27). However, similar effects have been reported in other species. In a prospective study of critically ill human patients, administration of 23.4% HS over 2–5 min resulted in a ≥20 mmHg decrease in systolic blood pressure in 13% of treated patients and systolic blood pressure < 90 mmHg in 16% of patients (28). In that study, the proposed mechanism was a direct effect of hypertonicity on vascular smooth muscle, resulting in arteriolar vasodilatation. The response observed in the present study is consistent with a vasodilatory effect of hypertonicity, potentially exacerbated by rapid administration of a high-concentration solution, although the specific haemodynamic mechanism cannot be determined from the available data.

In human patients, transient decreases in arterial pressure following 23.4% HS administration have not been considered clinically relevant. In anaesthetised horses, however, even transient decreases in MAP may be clinically important because hypotension, particularly MAP < 60–70 mmHg, is associated with post-anaesthetic myopathy and poorer recovery outcomes (29, 30). In the present study, MAP fell below 70 mmHg following HS injection in >60% of sample times and below 60 mmHg in >20% of sample times. These findings identify hypotension as the main safety concern for the use of repeated 23% HS boluses at the used volume and speeds in anaesthetised horses. Accordingly, repeated administration of 23% HS for EIT contrast enhancement cannot be recommended at this time, and future studies should evaluate whether lower concentrations, smaller volumes, or slower injection rates can provide adequate signal enhancement with less haemodynamic disturbance.

### 4.5 Changes over time

Repeated HS injections produced progressive increases in plasma sodium and chloride concentrations and marked increases in their fractional excretion, consistent with administration of a supraphysiologic NaCl load (16). By 60 min, mean plasma sodium values slightly exceeded recently defined arterial reference intervals, while chloride remained at the high end of normal (31). Plasma potassium concentrations decreased over time, however the lowest mean value observed (3.19 mmol L^−1^ at T60) remained within the reported reference range for horses (31). Although the final electrolyte concentrations were unlikely to cause clinical effects in healthy horses, peak transient changes in [Na^+^], [Cl^−^], and [K^+^ were not determined because electrolytes were not measured immediately post-injection. Further studies are needed to clarify the clinical relevance of these findings.

Pre-injection EELI and MAP also declined over the 60-min protocol. These changes may have been associated with repeated HS administration, but other time-dependent effects cannot be ruled out. The progressive increase in circulating electrolyte concentrations may have contributed to baseline impedance drift through increased thoracic conductivity. However, a gradual reduction in functional residual capacity during general anaesthesia, which would also reduce thoracic impedance, cannot be excluded (1). Concurrent diuresis and natriuresis following HS administration, potentially compounded by xylazine-associated diuresis, may have contributed to the progressive decrease in MAP; however, direct time-dependent effects of anaesthesia must also be considered (32). Further studies are required to clarify the mechanisms underlying baseline impedance drift and to determine whether repeated HS administration contributed to the progressive decrease in EELI. Regardless of the underlying mechanism, analytical approaches to repeated-bolus EIT data should account for progressive baseline drift.

### 4.6 Limitations, strengths, and future directions

These findings need to be interpreted in the context of the study design. The relatively small number of horses and lack of an *a priori* sample size calculation for this specific hypertonic saline analysis mean that the findings should be considered exploratory rather than definitive. This study was also performed opportunistically in conjunction with a study evaluating the effects of different airway management techniques on ventilation. While analysis did not find any confounding effect on the main outcome variables of this study, an effect cannot be completely discounted. A control group receiving an equivalent rapid intravenous bolus of isotonic saline was not included. Rapid intravenous boluses of saline are routinely administered during thermodilution cardiac output measurements in horses, though typically using smaller volumes of isotonic saline (approximately 20–40 ml for a 500 kg horse), and are not reported to cause haemodynamic instability (33). While the bolus volume used in the present study was larger, it still represents a small proportion of the circulating blood volume of an adult horse, however, in the absence of a control bolus, no conclusion can be drawn regarding the contribution of injection volume or the injection procedure itself. Another limitation of the present study is that detailed cardiovascular monitoring was not performed. Cardiac output, pulmonary arterial pressure, and echocardiographic parameters were not measured; therefore, the specific mechanisms underlying the observed haemodynamic responses cannot be determined. Arterial blood gases were collected intermittently during the study but not timed to capture the immediate post-injection period. Consequently, the peak effect of HS administration on gas exchange could not be reliably assessed.

This study has several notable strengths. To our knowledge, it is the first to demonstrate a consistent and reproducible drop in EELI in horses following administration of hypertonic saline, indicating that it can act as an enhancer for perfusion-related EIT signals in this species. The crossover design and standardisation of the anaesthetic protocol in both episodes of anaesthesia reduced inter-individual variability, and the reproducibility of the effect across different anaesthetic episodes strengthens confidence in the findings. A further strength is that all the horses were spontaneously breathing; this allowed detection of respiratory system effects that would have been missed under controlled ventilation.

This study is also the first to document haemodynamic alterations following the use of rapid HS administration for contrast enhancement. Hypertonic saline (3%−23%) has been used in other species for EIT contrast enhancement without apparent adverse effects, but cardiovascular responses were not systematically assessed in those studies. Consequently, the haemodynamic impact of HS-based EIT enhancement remains largely uncharacterised, and the hypotension observed in the present study should be considered an important safety finding. Given the growing interest in EIT for bedside monitoring and translational research, these findings may also have relevance for the development of HS-enhanced EIT protocols in human medicine.

As anaesthetised horses in the present study demonstrated a consistent decrease in MAP and frequent hypotension following rapid HS administration, these findings support targeted investigation to determine whether this response is species-specific, or protocol dependent. Future work should explore lower concentrations and/or volumes of HS than the 23% solution administered at 0.18 ml kg^−1^ in the present study. Concentrations in the range of approximately 5.85%−7% may represent a reasonable starting point, based on concentrations previously identified as optimal for EIT contrast enhancement in other species and those commercially available for clinical use (4). Future investigations should incorporate detailed assessment of immediate haemodynamic responses as well as targeted respiratory assessment to determine whether HS administration interferes with evaluation of ventilation and perfusion. In addition, study design and analysis should explicitly account for progressive baseline impedance drift when using repeated-bolus protocols, either by eliminating or adjusting for this effect.

## 5 Conclusion

This study demonstrates that bolus administration of 23% HS injected over 10 or 20 s produces consistent and reproducible decreases in EELI, supporting its potential utility as a conductive enhancer for perfusion-related EIT signal detection in anaesthetised horses. However, the frequent and clinically relevant reductions in MAP observed immediately following injection raise important safety concerns regarding repeated bolus administration of 23% HS in this species. Concurrent changes in TIV and breathing pattern may also complicate interpretation of ventilation-perfusion relationships derived from EIT. Accordingly, the use of repeated 23% HS boluses for EIT contrast enhancement in horses cannot be recommended at this time. The feasibility and safety of a single bolus injection require further investigation. Alternative strategies, including lower-concentration hyperosmolar contrast agents, should be explored as safer methods of enhancing EIT perfusion signals in horses.

## Data Availability

The raw data supporting the conclusions of this article will be made available by the authors, without undue reservation.
